# Interleukin-7 Modulates Anti-Tumor CD8^+^ T Cell Responses via Its Action on Host Cells

**DOI:** 10.1371/journal.pone.0159690

**Published:** 2016-07-22

**Authors:** Katrin Deiser, Diana Stoycheva, Ute Bank, Thomas Blankenstein, Thomas Schüler

**Affiliations:** 1 Institute of Molecular and Clinical Immunology, Medical Faculty, Otto-von-Guericke University, 39120 Magdeburg, Germany; 2 Institute of Immunology, Charité-Universitaetsmedizin Berlin, Campus Benjamin Franklin, 12200 Berlin, Germany; 3 Max-Delbrück-Center for Molecular Medicine, 13125 Berlin, Germany; Ohio State University, UNITED STATES

## Abstract

The adoptive transfer of antigen-specific CD8^+^ T cells is a promising approach for the treatment of chronic viral and malignant diseases. In order to improve adoptive T cell therapy (ATT) of cancer, recent strategies aim at the antibody-based blockade of immunosuppressive signaling pathways in CD8^+^ T cells. Alternatively, adjuvant effects of immunostimulatory cytokines might be exploited to improve therapeutic CD8^+^ T cell responses. For example, Interleukin-7 (IL-7) is a potent growth, activation and survival factor for CD8^+^ T cells that can be used to improve virus- and tumor-specific CD8^+^ T cell responses. Although direct IL-7 effects on CD8^+^ T cells were studied extensively in numerous models, the contribution of IL-7 receptor-competent (IL-7R^+^) host cells remained unclear. In the current study we provide evidence that CD8^+^ T cell-mediated tumor rejection in response to recombinant IL-7 (rIL-7) therapy is strictly dependent on IL-7R^+^ host cells. On the contrary, CD8^+^ T cell expansion is independent of host IL-7R expression. If, however, rIL-7 therapy and peptide vaccination are combined, host IL-7R signaling is crucial for CD8^+^ T cell expansion. Unexpectedly, maximum CD8^+^ T cell expansion relies mainly on IL-7R signaling in non-hematopoietic host cells, similar to the massive accumulation of dendritic cells and granulocytes. In summary, we provide evidence that IL-7R^+^ host cells are major targets of rIL-7 that modulate therapeutic CD8^+^ T cell responses and the outcome of rIL-7-assisted ATT. This knowledge may have important implications for the design and optimization of clinical ATT protocols.

## Introduction

The size of the peripheral T cell pool is remarkably stable throughout life. Although infections can cause a strong increase in T cell numbers, they usually return to steady-state levels after pathogen clearance. This indicates that self-regulatory mechanisms maintain T cell numbers [1]. A central factor controlling peripheral T cell homeostasis is IL-7. It acts as a growth and survival signal for T cells, which express the IL-7R and constitutively consume IL-7 [2]. Consequently, the size of the peripheral T cell pool becomes self-limiting as soon as IL-7 production and consumption reach an equilibrium [1]. Due to the lack of IL-7 consumption by T cells, IL-7 availability is increased in lymphopenic humans [3] and mice [4]. Lymphopenia-associated IL-7 overabundance contributes to the activation of naïve T cells, which undergo homeostatic or lymphopenia-induced proliferation (LIP) and convert into memory-like cells, which express high levels of CD44 and IFNγ [5].

The adoptive transfer of antigen-specific T cells is an important therapeutic option for the treatment of viral infections and cancer and has been performed successfully in animal models as well as in the clinic [6,7]. It is well established that the efficacy of adoptive T cell therapy (ATT) can be improved if recipient T cells are depleted by chemotherapy or irradiation prior to T cell transfer [6,8]. This positive effect of lymphodepletion results from the increased availability of T cell growth and survival factors such as IL-7 [9,10].

From our own experiments we know that thymus, lymph nodes, skin and intestine are the major sources of IL-7 in the mouse [11,12]. Nevertheless, steady-state IL-7 production is not sufficient for effective anti-tumor T cell responses under non-lymphopenic conditions. The injection of recombinant IL-7 (rIL-7) circumvents this problem and boosts anti-tumor T cell responses [13,14]. Since IL-7 promotes T cell survival [15,16], activation [17,18], proliferation [19] and memory T cell (T_M_) formation [20] its direct action on T cells is supposed to be the major cause for its potent anti-tumor effects [21]. For the effective treatment of viral infections and cancer by ATT high numbers of adoptively transferred CD8^+^ cells are required *in vivo* [7]. Their longevity and subsequent accumulation can be improved by rIL-7 therapy suggesting that this approach can be used to improve ATT [21]. Importantly, the adjuvant effects of rIL-7 correlate with tumor growth delay rather than complete rejection [13,22,23]. Given that i) regulatory immune cells such as dendritic cells (DCs) and granulocytes expand in response to elevated IL-7 levels [24,25] and ii) non-hematopoietic cells such as fibroblasts and intestinal epithelial cells express functional IL-7 receptors (IL-7R) [12,26], we hypothesized that IL-7R^+^ host cells might modulate anti-tumor CD8^+^ T cell responses.

In the current study we asked whether and how host IL-7R signaling affects ATT efficacy. For this purpose we established an ATT model, which enabled us to discriminate between direct and indirect effects of rIL-7 therapy on tumor-specific CD8^+^ T cells. Our data demonstrate, that LIP of CD8^+^ T cells and subsequent T_M_ differentiation are promoted by rIL-7 in a host IL-7R-independent manner. However, tumor rejection strictly requires host IL-7R expression. Furthermore, we show that IL-7R^+^ non-hematopoietic host cells are crucial for maximum CD8^+^ T cell expansion and T_M_ differentiation if rIL-7 therapy is combined with peptide vaccination. Importantly, despite efficient CD8^+^ T cells expansion, peptide vaccination deteriorates rIL-7-dependent ATT efficacy. In summary, we provide evidence that host IL-7R signaling modulates multiple aspects of CD8^+^ T cells activation and T_M_ differentiation and can promote tumor rejection in a context-dependent fashion.

## Materials and Methods

### Mice

C57BL/6J (WT), B6.PL-Thy1a/Cy (CD90.1^+^), B6.SJL-*Ptprc*^*a*^ *Pepc*^*b*^/BoyJ (CD45.1^+^), B6.129S7-Rag1^tm1Mom^/J (Rag^-/-^), B6.129S7-Il7r^tm1Imx^/J (IL-7R^-/-^), C57BL/6-Tg(TcraTcrb)1100Mjb/J (OT-I) (expressing a transgenic TCR specific for the chicken ovalbumin (OVA)-derived, H2-K^b^–restricted peptide OVA_257-264_ (SIINFEKL)), IL-7GCDL [11] and ChRluc mice [27] were bred in our animal facilities. All mice were housed under specific pathogen-free conditions. Animal experiments were performed according to institutional guidelines and were approved by the Landesamt für Gesundheit und Soziales Berlin (Permit Number: G0170/08) and Landesverwaltungsamt Sachsen-Anhalt (Permit Number: 2–1155 Uni MD).

### Generation of bone marrow (BM) chimeras and bioluminescence detection

BM recipients were anesthetized (Ketamin/Rompun i.p.), irradiated lethally and injected with BM cells i.v. 6–18 hours later. Donor BM was isolated from femur and tibia. BM from one donor was used to reconstitute 3 recipients. BM chimeras received antibiotics via the drinking water for 3–4 weeks and were used for experiments earliest 6 weeks after BM injection. To visualize luciferase activity in live animals, bioluminescence intensities (BLI) were measured using the IVIS Imaging system (Xenogen) as described before [11,27].

### Adoptive T cell transfer, peptide vaccination and IL-7 treatment

Naïve CD8^+^ T cells were purified from spleen and lymph nodes of the respective donor mice using CD8α-specific microbeads and AutoMACS (Miltenyi Biotec). 7 x 10^5^–1 x 10^6^ CD8^+^ T cells (purity >97%) were injected i.v. into the tail vein of recipient mice. For peptide vaccination, 50 μg of SIINFEKL were injected i.v. one day after T cell transfer. Control animals were injected with Dulbecco’s PBS. Prior to injection, recombinant murine IL-7 (rIL-7) (eBioscience) and anti-IL-7 mAb (M25; BioXCell) were mixed and incubated for 20 min at RT. Unless otherwise stated, 200 μl of 1,5 μg rIL-7 and 10 μg anti-IL-7 mAb in PBS were injected i.p every 3–4 days starting one day prior to T cell transfer.

### Tumor cell challenge

EG7 lymphoma cells produce chicken ovalbumin (OVA) and are targets of CD8^+^ OT-I T cells. EG7 cells were cultured in RPMI+10% FCS medium with 0.4 mg/ml G418. 1 x 10^6^ cells were injected s.c. in the right flank of the indicated mice. Mice with tumors >250 mm^3^ were scored as tumor positive. Tumor growth was monitored every 2–3 days. Mice were euthanized by cervical dislocation when tumors reached a diameter of 10–15 mm or when showing the following signs: hunched posture, inactivity, worsening body condition, rough coat, orbital tightening or abnormal breathing. Body weight was not assessed in this study.

### Flow cytometry

The following antibodies were used: anti-CD8α (53–6.7; eBioscience/Biolegend/BD), -CD90.1 (OX-7; Biolegend/BD), -CD62L (MEL-14; Biolegend/BD), -CD44 (IM7; eBioscience/Biolegend/BD), -KLRG-1 (2F1; eBioscience), PD-1 (J43; eBioscience), -Ki67 (SolA15; eBioscience), CD127 (A7R34; BD/Biolegend), CD132 (TUGm2; BD), -Bcl-2 (10C4; Biolegend), -IFN-γ (XMG1.2; Biolegend/eBioscience), -TNF-α (MP6-XT22; BD), -CD11c (N418; BD/Biolegend), -CD11b (MI70; BD/Biologend), Gr-1 (RB6-8-C5; Biolegend). Stimulation of CD8^+^ OT-I T cells with SIINFEKL and subsequent intracellular cytokine staining was performed as described recently [28]. Samples were measured on FACSCalibur, FACSCanto or LSRII flow cytometers and analyzed by FlowJo software (FlowJo, LLC).

### Statistical analysis

Statistical analysis and graphical representations were done using Prism5 software (GraphPad Software). Statistical significance was determined using a non-parametric two-tailed Mann-Whitney, paired Student’s t, 1- and 2-way Anova, log-rank or Wilcoxon matched-pairs signed rank test. * p<0,05; ** p<0,01; *** p<0,001.

## Results

### Host IL-7R signaling is crucial for rIL-7-dependent, CD8^+^ T cell-mediated tumor rejection

**Fig 1:**
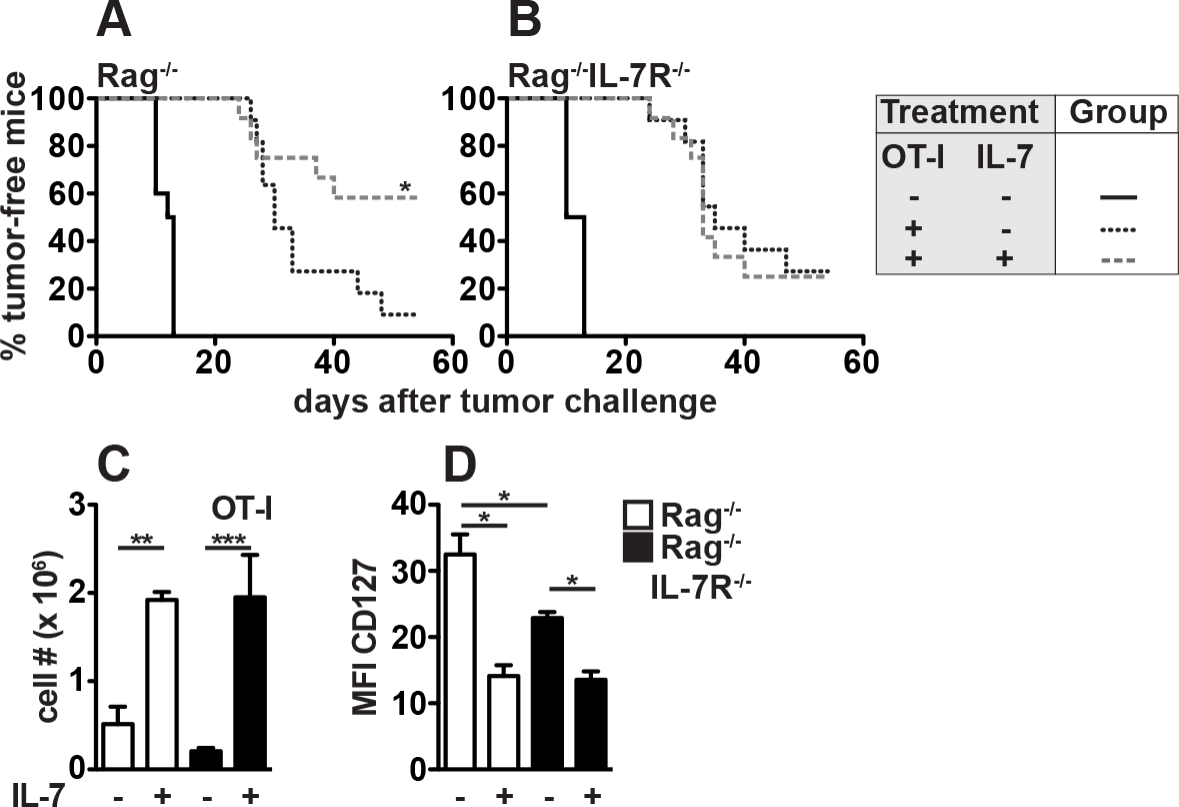
Host IL-7R signaling is required for CD8^+^ T cell-mediated tumor rejection in response to IL-7 treatment. (A-C) Rag^-/-^ and Rag^-/-^IL-7R^-/-^ mice were reconstituted with 7–10 x 10^5^ CD8^+^CD90.1^+^ OT-I T cells or were left untreated (+/- OT-I). OT-I-reconstituted mice received rIL-7 (+ IL-7) or PBS (- IL-7) every 3–4 days for 18 days starting one day before T cell transfer. (A, B) 22–23 days after T cell transfer, some mice were challenged s.c. with 1 x 10^6^ ovalbumin-expressing EG7 lymphoma cells. Mice with tumors >250 mm^3^ were scored as tumor positive. Pooled data from 2 independent experiments with a total of 10–12 mice per group are shown. Statistical significance was calculated using the log-rank test. (C, D) Some recipients were not challenged with EG7 but analyzed for OT-I expansion and phenotype. (C) Splenic CD8^+^CD90.1^+^ OT-I T cells were quantified 21–25 days after adoptive transfer. Pooled data (±SEM) from 2 independent experiments with a total of 7–9 mice/group are shown. (D) CD127 expression of splenic OT-I T cells was determined 5 days after adoptive transfer. Data (±SEM) for 3–4 mice/group are shown.

Naïve CD8^+^ T cells transferred into lymphopenic mice undergo lymphopenia-induced proliferation (LIP), differentiate into CD44^hi^ memory-like T cells [5] and limit tumor growth [8]. Accordingly, naive CD8^+^ TCR-transgenic (tg) OT-I T cells specific for the chicken ovalbumin (OVA)-derived, H2-K^b^-restricted peptide SIINFEKL, expanded and up regulated CD44 in lymphopenic Rag1-deficient (Rag^-/-^) recipients within 21–27 days after transfer. Expansion and T_M_ differentiation were not observed in OT-I-reconstituted, non-lymphopenic wildtype (WT) mice (S1 Fig). Whether LIP-associated CD8^+^ T cell activation leads to tumor growth inhibition was tested next. Twenty-two days after reconstitution with naive CD8^+^ OT-I T cells, Rag^-/-^ mice were challenged s.c. with 10^6^ OVA-expressing EG7 lymphoma cells. EG7 tumors grew rapidly in untreated Rag^-/-^ mice while adoptive T cell transfer strongly delayed tumor growth (Fig 1A). Nevertheless, only 1 mouse out of 11 remained long-term tumor-free.

In order to investigate whether IL-7 therapy promotes tumor rejection, groups of OT-I-reconstituted Rag^-/-^ mice received rIL-7 every 3–4 days for 18 days starting one day prior to adoptive T cell transfer. To improve its function, rIL-7 was complexed with IL-7-specific antibodies (αIL-7) prior to injection as described before [29]. 7/12 Rag^-/-^ mice receiving OT-I T cells plus IL-7 therapy completely rejected EG7 lymphoma cells demonstrating that IL-7 therapy strongly enhances OT-I-dependent tumor rejection in our experimental system (Fig 1A). Importantly, rIL-7 treatment did not affect primary EG7 growth in either host (data not shown).

Several studies provided evidence that rIL-7 promotes activation, survival, function of CD8^+^ T cells [15–19,30] and memory T cell (T_M_) formation [20]. So far, however, these effects were considered to result from direct effects of rIL-7 on CD8^+^ T cells. Besides CD8^+^ T cells, however, numerous hematopoietic and non-hematopoietic cells express the IL-7 receptor (IL-7R) [31]. Hence, it remained unclear whether the success of IL-7-assisted adoptive T cell transfers relies on IL-7R signaling in CD8^+^ T cells and/or host cells. In order to address this question, Rag^-/-^ mice lacking the IL-7Rα chain (Rag^-/-^IL-7R^-/-^) were reconstituted with OT-I T cells, received IL-7 therapy or PBS and were challenged with EG7 lymphoma cells. This approach allowed us to separate direct from indirect effects of rIL-7 on CD8^+^ T cell-mediated lymphoma rejection. As compared to untreated controls, OT-I cells strongly delayed tumor growth in Rag^-/-^IL-7R^-/-^ recipients (Fig 1B) similar to what we had observed in Rag^-/-^ mice (Fig 1A). This demonstrates that host IL-7R-deficiency does not limit LIP-associated CD8^+^ T_M_ differentiation (S1 Fig) and subsequent anti-tumor immunity (Fig 1B). In contrast to Rag^-/-^ mice, rIL-7 treatment of OT-I-reconstituted Rag^-/-^IL-7R^-/-^ mice did not improve anti-tumor CD8^+^ T cell responses (Fig 1B) indicating that direct effects of rIL-7 on CD8^+^ T cells are not sufficient for successful tumor rejection. In fact, IL-7R signaling by host cells is crucial for tumor rejection after CD8^+^ T cell transfer and rIL-7 therapy. Next we studied whether impaired tumor rejection in Rag^-/-^IL-7R^-/-^ mice resulted from reduced LIP. Rag^-/-^ and Rag^-/-^IL-7R^-/-^ mice were reconstituted with CD8^+^ OT-I T cells and spleen cells were analyzed 21–27 days later. CD44 levels (S1A Fig) and recovery rates (S1B Fig) did not differ between OT-I cells from Rag^-/-^ and Rag^-/-^IL-7R^-/-^ mice. In conclusion, LIP-driven expansion and T_M_ formation are independent of host IL-7R signaling.

To analyze the impact of host IL-7R signaling on rIL-7 therapy-related T_M_ differentiation and expansion, Rag^-/-^ and Rag^-/-^IL-7R^-/-^ mice were reconstituted with OT-I T cells and treated with rIL-7 (+IL-7) or PBS (-IL-7) as described above. In accordance with S1B Fig, similar numbers of OT-I cells were recovered from spleens of PBS-treated Rag^-/-^ and Rag^-/-^IL-7R^-/-^ mice 21–25 days after adoptive transfer (Fig 1C). IL-7 signaling suppresses IL-7Rα chain (CD127) expression by naive T cells [32]. Accordingly, a strong and host-independent down-modulation of CD127 was observed 5 days after adoptive transfer and rIL-7 treatment (Fig 1D). Importantly, OT-I T cells expressed less CD127 in PBS-treated Rag^-/-^IL-7R^-/-^ than in Rag^-/-^ mice arguing for elevated steady state IL-7 levels in the latter. Nevertheless, this did not affect the long-term abundance (Fig 1C) or early rIL-7 responsiveness of OT-I T cells (Fig 1D).

In conclusion, host IL-7R signaling is dispensable for rIL-7-driven CD8^+^ T cell expansion (Fig 1C) but not for subsequent tumor rejection. This highlights that high numbers of therapeutic CD8^+^ T cells do not necessarily correlate with therapeutic success.

### Host IL-7R signaling is not required for rIL-7-induced T_M_ differentiation but for granulocyte and DC expansion

**Fig 2:**
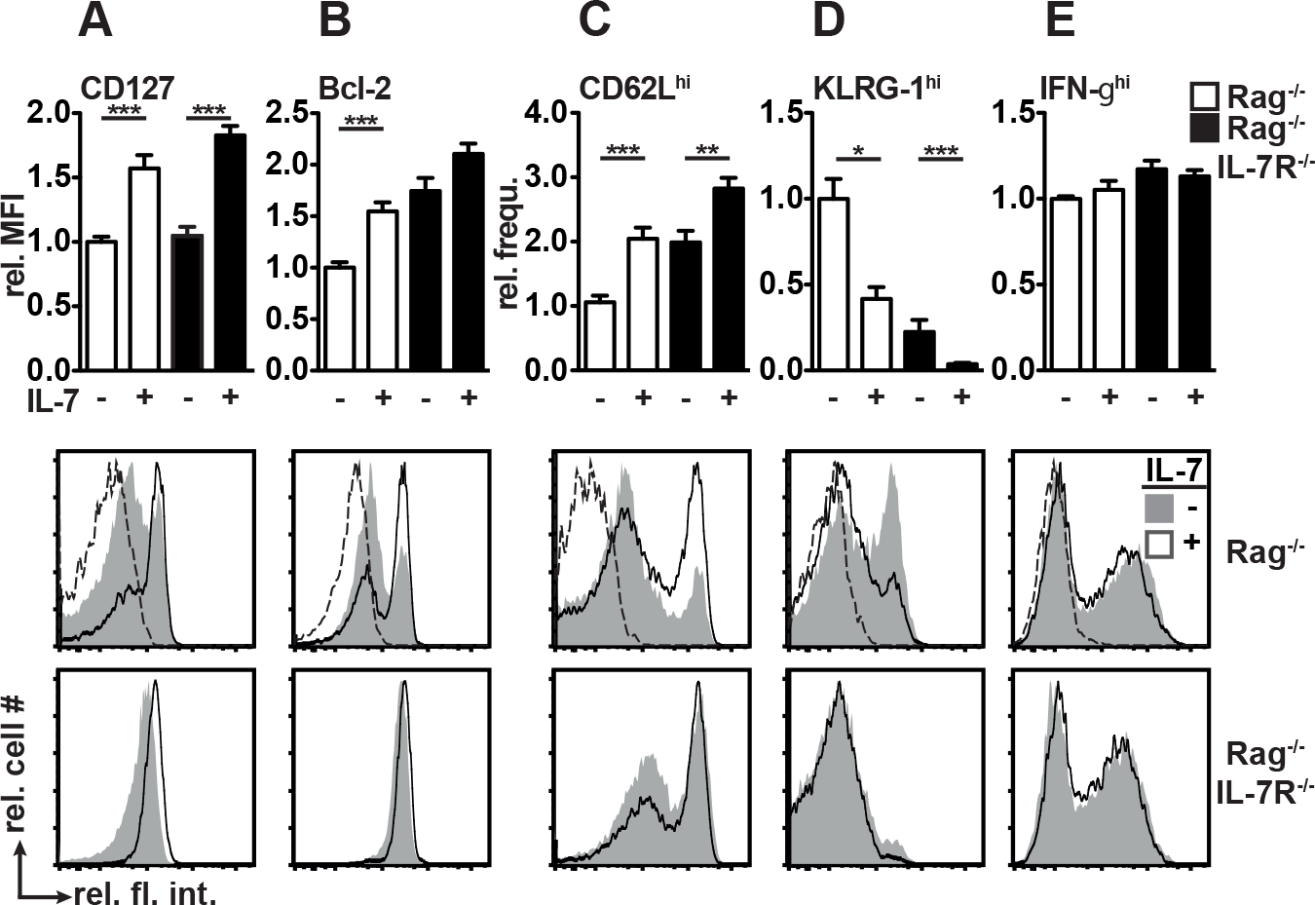
Host IL-7R signaling is not required for rIL-7-induced T_M_ differentiation. (A-E) Rag^-/-^ and Rag^-/-^IL-7R^-/-^ mice were treated with rIL-7 or PBS (+/- IL-7) every 3–4 days starting one day prior to adoptive transfer of 1 x 10^6^ CD8^+^CD90.1^+^ OT-I T cells. 21–25 days after transfer, splenic OT-I T cells were analyzed by flow cytometry. After gating on CD8^+^CD90.1^+^ or CD8^+^Vα2^+^ OT-I T cells, mean fluorescence intensities (MFIs) for (A) CD127 and (B) Bcl-2 as well as the frequencies (frequ.) of (C) CD62L^hi^, (D) KLRG-1^hi^ and (E) IFN-γ^hi^ cells were determined. IFN-γ production was measured after *in vitro* re-stimulation with 1 μM SIINFEKL peptide in the presence of brefeldin A for 6 hours. (A-E) Data shown in bar diagrams were normalized to the mean values determined in PBS-treated Rag^-/-^ mice and are shown as relative (rel.) values. Pooled data (±SEM) from 2–3 independent experiments with a total of 7–13 mice/group are shown. Histogram overlays show relative cell numbers (rel. cell #) and relative fluorescence intensities (rel. fl. int.) for individual mice after gating on OT-I cells. Dashed lines in overlays represent (A-D) FMO control samples or (E) stained cells without prior SIINFEKL stimulation.

CD8^+^ T_M_ express high levels of CD127 and the anti-apoptotic molecule B cell lymphoma protein-2 (Bcl-2) [33], which can be up regulated by IL-7 [34]. In PBS-treated Rag^-/-^ mice CD127^hi^ and Bcl-2^hi^ OT-I T_M_ were less frequent than in Rag^-/-^IL-7R^-/-^ mice (Fig 2A and 2B). In response to rIL-7 treatment, however, resulting OT-I T_M_ expressed elevated levels of CD127 and Bcl-2 in both hosts 21–25 days after transfer (Fig 2A and 2B). Hence, despite early CD127 down-regulation (Fig 1D), IL-7 therapy promoted the generation of CD127^hi^Bcl-2^hi^ OT-I T_M_ irrespective of the host. IL-7 therapy also promoted the generation of CD62L^hi^ OT-I cells (Fig 2C) expressing low levels of KLRG-1 (Fig 2D). Furthermore, a high percentage of OT-I cells recovered from both hosts rapidly produced IFN-γ after short-term *in vitro* re-stimulation (Fig 2E). Hence, rIL-7 therapy favors the generation of functional CD8^+^ T_M_ cells with a CD127^hi^Bcl-2^hi^CD62L^hi^KLRG-1^lo^ phenotype in a host-independent fashion. Interestingly, despite their high numbers (Fig 1C) and favorable phenotype (Fig 2), rIL-7-induced OT-I T_M_ cells failed to reject tumors in Rag^-/-^IL-7R^-/-^ mice (Fig 1B). This suggested a contribution of rIL-7-responsive host cells to EG7 elimination.

**Fig 3:**
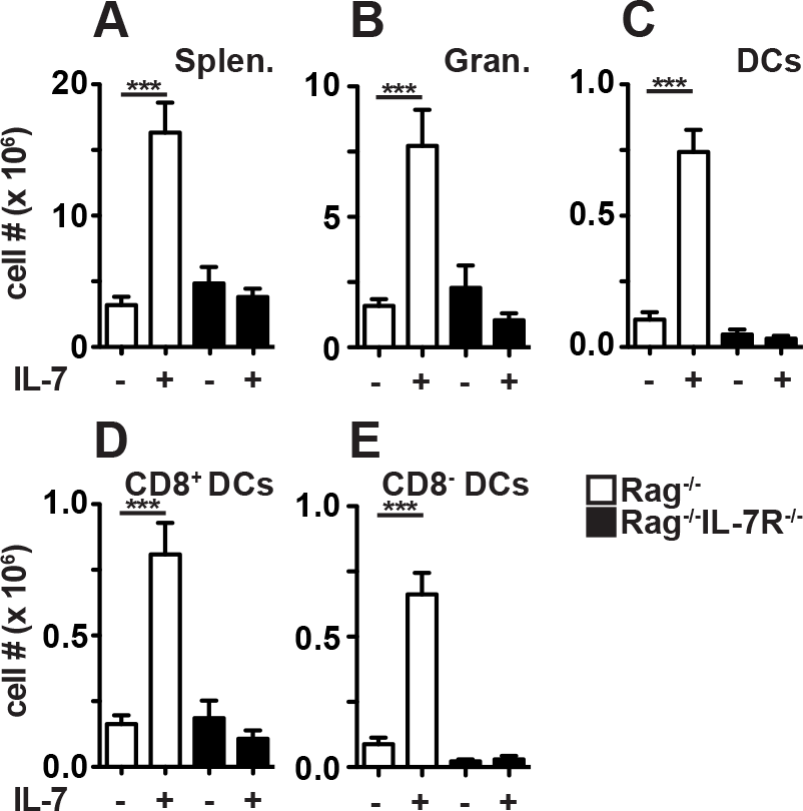
Host IL-7R signaling is required for granulocyte and DC expansion in response to rIL-7. Rag^-/-^ and Rag^-/-^IL-7R^-/-^ mice were treated with rIL-7 or PBS (+/- IL-7) every 3–4 days and spleens were analyzed by flow cytometry after 10–24 days. (A-E) Shown are numbers of (A) splenocytes (Splen.), (B) CD11b^+^Gr1^+^ granulocytes (Gran.), (C) CD11c^+^MHC-II^+^ dendritic cells (DCs), (D) CD8^+^ and (E) CD8^-^ DCs. Shown are pooled data (±SEM) from 2 independent experiments with a total of 7–8 mice/group.

After rIL-7 treatment, spleen cell numbers increased dramatically in Rag^-/-^ mice (Fig 3A). In agreement with previous studies [24,25], CD11b^+^Gr-1^+^ granulocytes and CD11c^+^MHCII^+^ DCs expanded most efficiently in response to rIL-7 (Fig 3B and 3C). Among the latter, CD8^+^ lymphoid and CD8^-^ myeloid DCs responded similarly well (Fig 3D and 3E). In Rag^-/-^IL-7R^-/-^ mice rIL-7 therapy failed to induce the expansion of splenocytes, granulocytes and DCs (Fig 3A–3E) excluding IL-7R-independent side effects of our treatment regimen. In summary, host IL-7R signaling is crucial for rIL-7-induced, CD8^+^ T cell-mediated tumor rejection (Fig 1A and 1B), is not essential for CD8^+^ T_M_ differentiation (Fig 2) but promotes the expansion of granulocytes and DCs.

### Host IL-7R signaling promotes CD8^+^ T cell expansion and modulates T_M_ differentiation in response to peptide vaccination and IL-7 therapy

**Fig 4:**
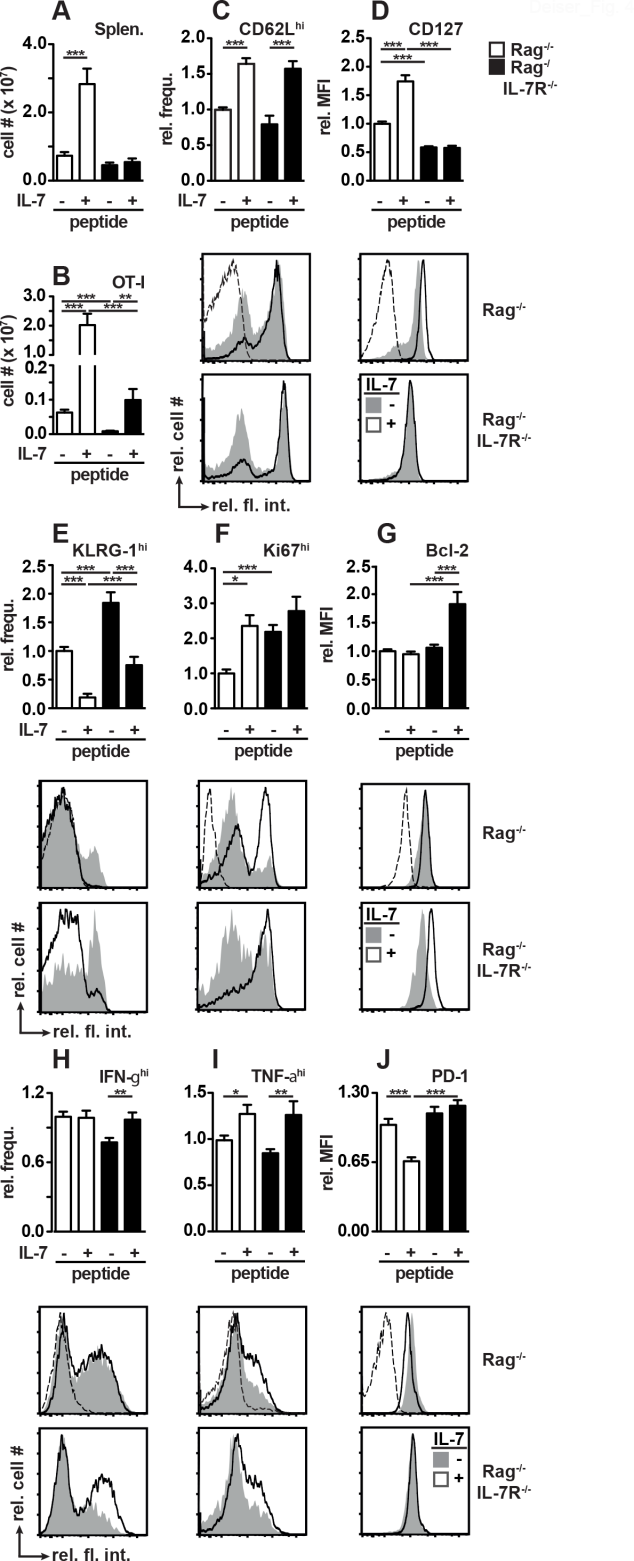
Host IL-7R signaling modulates CD8^+^ T cell expansion and differentiation in response to peptide vaccination and IL-7 therapy. (A-J) Rag^-/-^ and Rag^-/-^IL-7R^-/-^ mice were reconstituted with CD8^+^CD90.1^+^ OT-I T cells and treated with SIINFEKL +/- rIL-7 as described in Fig 4. Three weeks after T cell transfer (A, B) spleens and (C-J) peripheral blood were analyzed by flow cytometry. Shown are numbers of (A) splenocytes and (B) splenic CD8^+^CD90.1^+^ OT-I T cells. After gating on OT-I T cells, relative frequencies (frequ.) of (C) CD62L^hi^, (E) KLRG-1^hi^, (F) Ki67^hi^, (H) IFN-γ^hi^, (I) TNF-α^hi^ OT-I cells and relative MFIs for (D) CD127, (Γ) Bcl-2 and (J) PD-1 were determined. (H, I) Cytokine production was measured after *in vitro* stimulation of PBMCs with 1 μM SIINFEKL peptide in the presence of Rag^-/-^ splenocytes, brefeldin A and monensin for 6 hours. Relative (rel.) values were calculated as described in Fig 2. Bar diagrams show pooled data (±SEM) from 3 independent experiments with a total of 15–18 mice/group. Histogram overlays show representative results for individual mice. Dashed lines in overlays represent (C-G and J) FMO control samples or (H, I) stained cells without prior SIINFEKL stimulation.

IL-7 administration improves vaccination-induced T cell responses [13,22]. To test whether this also requires host IL-7R signaling, Rag^-/-^ and Rag^-/-^IL-7R^-/-^ mice were reconstituted with OT-I cells, immunized with SIINFEKL and treated with rIL-7. Peptide-vaccinated mice receiving PBS served as controls. As shown in Fig 4A rIL-7 treatment strongly increased spleen cell numbers in Rag^-/-^ but not Rag^-/-^IL-7R^-/-^ mice. Furthermore, rIL-7 treatment promoted OT-I cell expansion in Rag^-/-^ mice (Fig 4B). Although to a much lesser extent, this was also observed in rIL-7-treated Rag^-/-^IL-7R^-/-^ mice (Fig 4B). Thus, IL-7R signaling in CD8^+^ T cells promotes some degree of rIL-7-induced CD8^+^ T cell expansion although the full-blown response requires host IL-7R expression.

To test whether IL-7R^+^ host cells also modulate T_M_ differentiation the phenotype of peripheral blood OT-I cells was determined by flow cytometry. In Rag^-/-^ and Rag^-/-^IL-7R^-/-^ mice, frequencies of CD62L^hi^ OT-I cells were similar after vaccination (Fig 4C). This was further increased by rIL-7 and was independent of host IL-7R expression (Fig 4C). OT-I cells expressed similar levels of CD127 after vaccination of Rag^-/-^ and Rag^-/-^IL-7R^-/-^ mice. To our surprise, rIL-7 therapy further promoted CD127 expression by OT-I cells only in Rag^-/-^ mice (Fig 4D). KLRG-1^hi^ OT-I cells were more frequent in vaccinated Rag^-/-^IL-7R^-/-^ than in Rag^-/-^ mice. In both hosts their abundance decreased in response to rIL-7, though to a lesser extent in Rag^-/-^IL-7R^-/-^ mice (Fig 4E). After vaccination, OT-I proliferation was lower in Rag^-/-^ mice as shown by low numbers of Ki67^hi^ cells (Fig 4F). Bcl-2 expression was similar in vaccinated Rag^-/-^ and Rag^-/-^IL-7R^-/-^ mice and was up regulated by rIL-7 only in the latter (Fig 4G). In summary, the rIL-7-induced up-regulation of CD62L by OT-I cells was largely independent of IL-7R^+^ host cells. In contrast, host cells promoted the expansion of OT-I cells (Fig 4B) and modulated their expression of CD127, KLRG-1 and Bcl-2 in response to rIL-7 therapy (Fig 4D, 4E and 4G).

After vaccination, OT-I cells isolated from Rag^-/-^ mice produced high levels of IFN-γ (Fig 4H). This response was less pronounced in Rag^-/-^IL-7R^-/-^ mice but could be improved by rIL-7 treatment (Fig 4H). Importantly, rIL-7 treatment could not further promote the generation of IFN-γ^hi^ OT-I cells in Rag^-/-^ mice (Fig 4H). OT-I cells producing high levels of TNF-α were similarly frequent in Rag^-/-^ and Rag^-/-^IL-7R^-/-^ mice and further expanded in both hosts in response to rIL-7 treatment (Fig 4I). The expression of PD-1, a marker for dysfunctional T cells, was comparable for OT-I cells recovered from mice of both strains. In agreement with a previous study [35], rIL-7 administration reduced PD-1 expression on OT-I cells in Rag^-/-^ mice (Fig 4J). Surprisingly, this was not the case in Rag^-/-^IL-7R^-/-^ mice (Fig 4J) indicating that IL-7R^+^ host cells determine the levels of PD-1 expression by CD8^+^ T cells. Hence, immunomodulation by rIL-7 relies on complex interactions between IL-7R^+^ host and therapeutic CD8^+^ T cells.

### IL-7R^+^ non-hematopoietic cells are crucial for CD8^+^ T cell expansion in response to vaccination and rIL-7 therapy

Prolonged exposure to elevated levels of IL-7 down modulates *il-7* gene activity in non-BM-derived cells [4,12], which are the major source of IL-7 *in vivo* [20]. To determine whether non-hematopoietic cells respond to rIL-7 therapy, IL-7 reporter mice expressing luciferase under the control of the *il-7* promoter [11] were treated with rIL-7. As a readout for *il-7* gene activity, bioluminescence intensities (BLI) were determined before and after rIL-7 treatment. As shown in S2 Fig, rIL-7 administration reduced *il-7* promoter activity significantly demonstrating that IL-7-producing non-BM-derived cells indeed respond to rIL-7 therapy.

**Fig 5:**
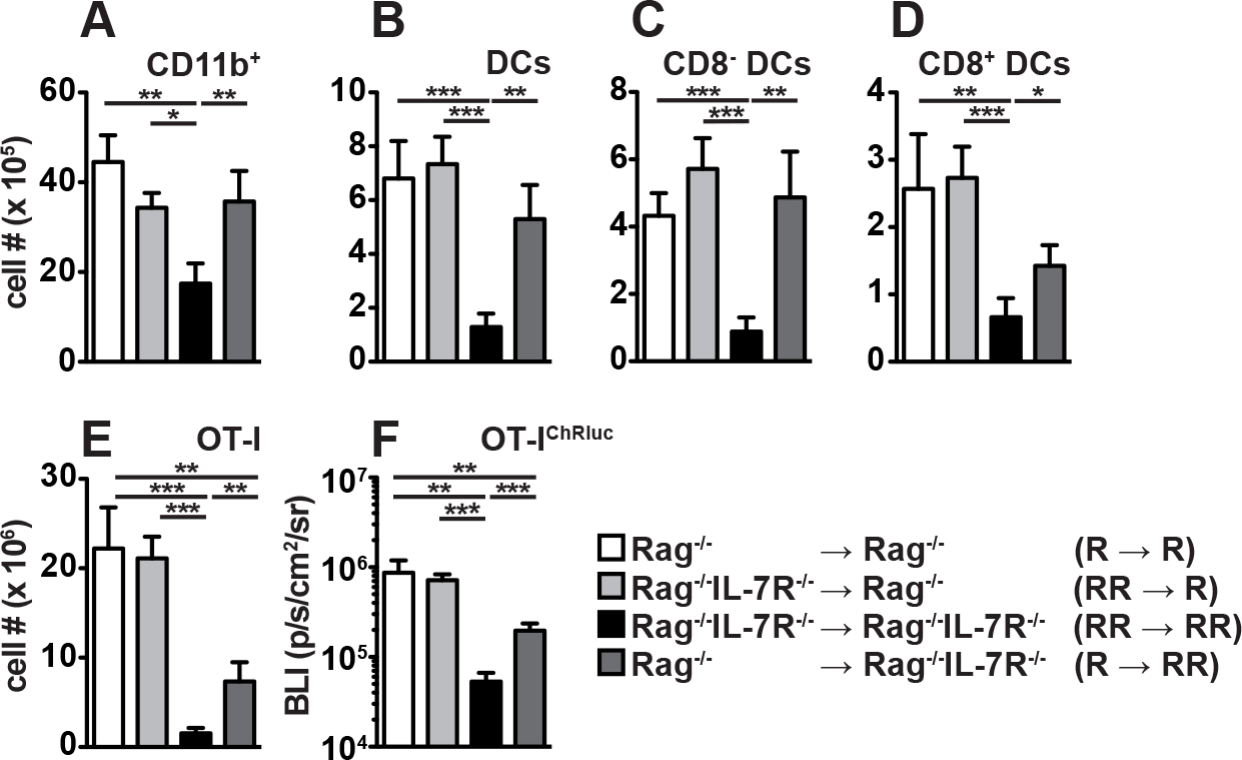
IL-7R signaling in non-hematopoietic host cells contributes to granulocyte, DC and CD8^+^ T cell expansion in response to IL-7 therapy. Lethally irradiated CD45.1^+^ Rag^-/-^ and CD45.2^+^ Rag^-/-^IL-7R^-/-^ mice were reconstituted with the indicated bone marrow (BM) cells (donor→recipient). After successful BM reconstitution, BM chimeras received 1 x 10^6^ CD8^+^ OT-I T cells. One day later, mice were vaccinated with 50 μg SIINFEKL. IL-7 treatment was done as described above. (A-E) Three weeks after T cell transfer, recipient spleens were analyzed by flow cytometry and the numbers of the indicated cell types were determined. Shown are pooled data (±SEM) of 3 independent experiments with a total of 10–17 mice/group. (F) BM chimeras received 1 x 10^6^ renilla luciferase-transgenic (ChRluc^tg^) CD8^+^ OT-I T cells (OT-I^ChRluc^) and were treated with SIINFEKL and rIL-7 as described above. Six days after T cell transfer, mice were treated with 100 μg colenterazine to determine bioluminescence intensities (BLI) as a readout for OT-I abundance and distribution. Shown are pooled results (±SEM) of 2 independent experiments with a total of 5–10 mice/group.

In order to study the relative contribution of IL-7R signaling in hematopoietic versus non-hematopoietic host cells to rIL-7-induced CD8^+^ T cell responses, we generated bone marrow (BM) chimeric mice. Lethally irradiated CD45.1^+^ Rag^-/-^ mice received BM from CD45.1^+^ Rag^-/-^ (R → R chimeras) or CD45.2^+^ Rag^-/-^IL-7R^-/-^ mice (RR → R chimeras). Furthermore, lethally irradiated CD45.2^+^ Rag^-/-^IL-7R^-/-^ mice received BM from CD45.2^+^ Rag^-/-^IL-7R^-/-^ (RR → RR chimeras) or CD45.1^+^ Rag^-/-^ mice (R → RR chimeras). Flow cytometric analysis of CD45.1/.2-disparate RR → R and R → RR chimeras revealed that donor BM contributed to the generation of around 97% of splenic CD11b^+^ cells (data not shown). BM-reconstituted mice received OT-I cells, vaccination and rIL-7 therapy as described above. Three weeks after adoptive T cell transfer, we first quantified splenic CD11b^+^ cells and DCs. To our surprise, IL-7R expression by BM-derived cells was dispensable for CD11b^+^ cell expansion if non-BM-derived cells expressed the IL-7R (Fig 5A; R → R vs RR → R chimeras). If, however, BM- and non-BM-derived cells lacked the IL-7R, CD11b^+^ cell expansion was strongly impaired (RR → RR). Only a partial recovery was observed if BM-derived cells produced the IL-7R (Fig 5A; R → RR) suggesting that IL-7R signaling in BM- and non-BM-derived cells synergize to promote therapy-induced CD11b^+^ cell expansion. Similarly, the expansion of IL-7R-competent and -deficient DCs was comparable in Rag^-/-^ BM recipients (R → R vs RR → R). On the contrary, DC expansion was strongly impaired in RR → RR chimeras and only partially recovered in R → RR chimeras (Fig 5B). Hence, IL-7R signaling in non-BM cells is crucial for therapy-associated DC expansion. However, this appeared to be more important for CD8^+^ DCs than for CD8^-^ DCs (Fig 5C and 5D).

Similar to DCs, OT-I accumulation in the spleen was independent of IL-7R expression by BM derived cells if non-BM-derived cells expressed the IL-7R (Fig 5E; R → R vs RR → R). In accordance with Fig 4B, OT-I responses were least efficient in chimeras lacking the IL-7R on BM-derived and non-BM-derived cells (Fig 5E; RR → RR). Importantly, IL-7R expression by BM-derived cells alone was insufficient to fully recover OT-I accumulation (Fig 5E; R → RR).

IL-7 treatment is known to alter homing patterns of CD8^+^ T cells [36]. To exclude migration-related differences in splenic OT-I cell numbers, BM chimeras were reconstituted with renilla luciferase-transgenic (ChRluc^tg^) CD8^+^ OT-I T cells [27], vaccinated and treated with rIL-7. Six days later, whole-body BLI levels were determined to quantify relative OT-I cell numbers in an unbiased, tissue-independent fashion. As shown in Fig 5F, ChRluc^tg^ OT-I were similarly abundant in R → R and RR → R chimeras. On the contrary, their frequency was strongly impaired in RR → RR chimeras and only partially recovered in R → RR chimeras. Hence, these results confirm Fig 5E and further emphasize the importance of IL-7R^+^ non-BM cells for rIL-7-induced CD8^+^ T cell expansion.

### IL-7R^+^ non-BM cells modulate CD8^+^ T_M_ differentiation in response to vaccination and rIL-7 therapy

**Fig 6:**
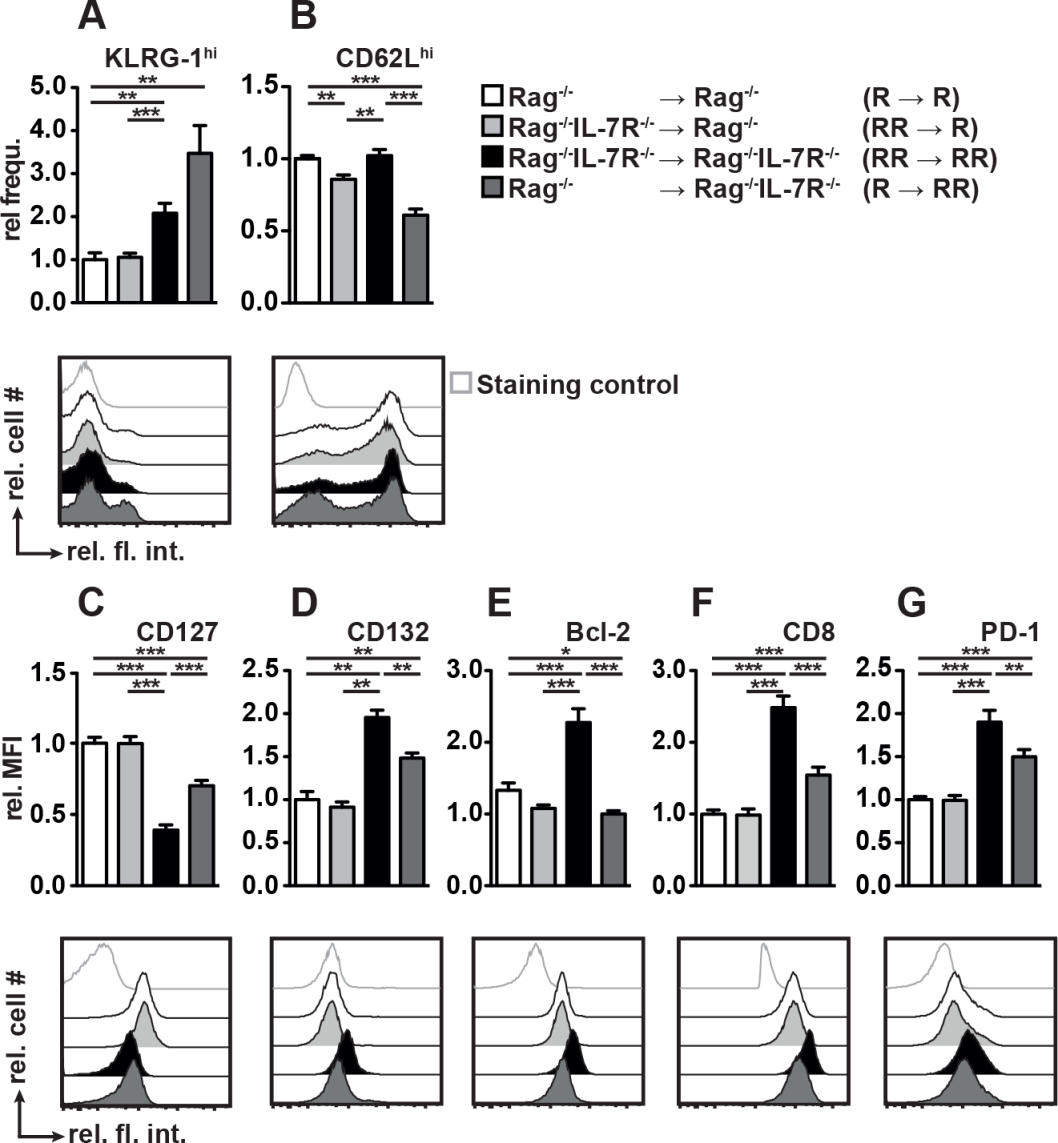
IL-7R signaling in non-hematopoietic cells regulates CD8^+^ T cell differentiation in response to peptide vaccination and IL-7 therapy. (A-G) The indicated BM chimeras received CD8^+^ OT-I T cells and were treated with SIINFEKL and rIL-7 as described above. Three weeks after T cell transfer, splenic CD8^+^ OT-I T cells were analyzed by flow cytometry. Bar diagrams show pooled results (±SEM) of 2–3 independent experiments with a total of (A, B, C, E, G) 10–17 or (D) 4–10 mice/group. Histogram overlays show representative results for individual mice. Grey-lined histograms represent FMO staining controls.

Having shown that non-BM cells promote the expansion of CD8^+^ T cells, we analyzed their phenotype. Three weeks after adoptive transfer, OT-I cells isolated from the spleens of BM chimeras were analyzed by flow cytometry. In agreement with Fig 4E, KLRG-1^hi^ OT-I cells were more frequent in RR → RR than in R → R chimeras (Fig 6A) further emphasizing that T_M_ differentiation into KLRG-1^hi^ cells is modulated by IL-7R^+^ host cells. These cells appear to be of BM and non-BM origin as shown by the fact that R → RR chimeras contained most KLRG-1^hi^ OT-I cells. However, their numbers were reduced in RR → RR chimeras and lowest in R → R and RR → R chimeras (Fig 6A).

The frequencies of CD62L^hi^ OT-I cells were identical in R → R and RR → RR chimeras suggesting their host IL-7R-independence (Fig 6B). However, CD62L^hi^ OT-I cells were less abundant in RR → R and R → RR (Fig 6B). This indicates that IL-7R^+^ BM- and non-BM-derived cells are part of a complex network exerting opposing functions on CD62L^hi^ T_M_ differentiation.

Highest levels of CD127 were found on OT-I cells from R → R and RR → R chimeras and lowest on those from RR → RR chimeras (Fig 6C) demonstrating that IL-7R expression by non-BM cells is a prerequisite for maximum CD127 expression by CD8^+^ T cells. If only BM-derived cells expressed the IL-7R (R → RR) CD127 levels were significantly higher than in RR → RR chimeras but still below those found on OT-I cells primed in RR → R chimeras (Fig 6C). Thus, IL-7R^+^ non-BM cells are major regulators of CD127 expression by OT-I cells.

The genes encoding Bcl-2 and CD8 are direct targets of IL-7 and other cytokines utilizing the IL-2Rγ_c_ (CD132) for signal transduction [37]. CD127^lo^ OT-I cells from RR → RR chimeras expressed highest levels of CD132, Bcl-2 and CD8 (Fig 6D–6F) suggesting that other CD132-dependent cytokines than IL-7 caused the differentiation of CD127^lo^CD132^hi^Bcl-2^hi^CD8^hi^ T_M_ cells in RR → RR chimeras. In contrast, R → R and RR → R chimeras rather contained CD127^hi^CD132^lo^Bcl-2^lo^CD8^lo^ OT-I cells (Fig 6C–6F). However, if only BM-derived cells expressed the IL-7R (R → RR) we observed an intermediate OT-I phenotype (Fig 6C–6G and 6F).

In response to rIL-7 treatment PD-1 was downregulated only on OT-I cells primed in Rag^-/-^ mice but not on those primed in Rag^-/-^IL-7R^-/-^ mice (Fig 4J). In agreement with this, PD-1 expression was most pronounced in RR → RR chimeras (Fig 6G). PD-1 levels were significantly lower in both groups of chimeras expressing IL-7R on non-BM-derived cells and (R → R and RR → R). In contrast, OT-I cells from R → RR chimeras showed an intermediate phenotype (Fig 6G) indicating that IL-7R^+^ non-BM-derived cells are major regulators of PD-1 expression by CD8^+^ T cells.

In summary, IL-7R^+^ host cells promoted the expansion of CD8^+^ T_M_ cells in response to rIL-7 therapy (Fig 4B–4J). Surprisingly, regulatory host cells were mainly of non-BM origin (Figs 5E, 5F and 6).

### The combination of rIL-7 therapy and peptide vaccination impairs T cell-dependent tumor rejection in Rag^-/-^ mice

**Fig 7:**
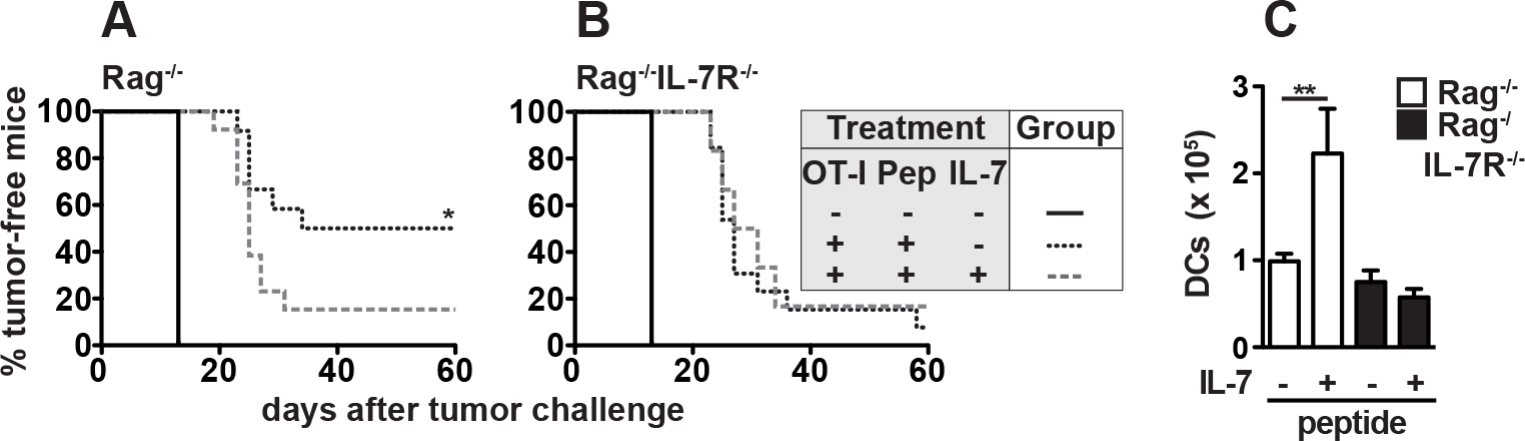
The combination of rIL-7 therapy and peptide vaccination impairs T cell-dependent tumor rejection in Rag^-/-^ mice. (A) Rag^-/-^ and (B) Rag^-/-^IL-7R^-/-^ mice were reconstituted with 1 x 10^6^ CD8^+^CD90.1^+^ OT-I T cells or were left untreated (+/- OT-I). One day later, OT-I-reconstituted mice were either vaccinated with 50 μg SIINFEKL or received PBS (+/- Pep). rIL-7 or PBS (+/- IL-7) were injected every 3–4 days for 19 days starting one day before T cell transfer. Mice were challenged s.c. with 1 x 10^6^ EG7 tumor cells three weeks after T cell transfer. Mice with tumors larger than 250 mm^3^ were scored as tumor positive. Shown are pooled data from 2 independent experiments with a total of 12–13 T cell reconstituted mice. Primary tumor growth was analyzed in untreated Rag^-/-^ and Rag^-/-^IL-7R^-/-^ mice (n = 3). Statistical significance was calculated using the log-rank test. (C) The numbers of splenic DCs were determined in tumor-bearing mice 28–37 days after tumor challenge. Pooled data (±SEM) from 2 independent experiments with a total of 6–11 mice/group are shown. Statistical significance was calculated using the Mann-Whitney test.

CD8^+^ CD62L^hi^KLRG-1^lo^IFN-γ^hi^PD-1^lo^ T_M_ cells are well suited to provide long-term protection against chronic infections and tumors [7,38,39]. Peptide vaccination and rIL-7 therapy induced the generation of such T_M_ cells in Rag^-/-^ mice (Fig 4B–4J), a process that was mainly controlled by IL-7R^+^ non-hematopoietic cells (Figs 5E, 5F and 6). However, their therapeutic potential remained unclear. To test this, Rag^-/-^ mice were reconstituted with CD8^+^ OT-I T cells and vaccinated with SIINFEKL one day later. Additionally, mice received rIL-7 or PBS according to the scheme described above. To ensure appropriate OT-I expansion and differentiation, mice were challenged with EG7 cells 21 days after adoptive T cell transfer. In untreated Rag^-/-^ control mice, EG7 tumors grew rapidly (Fig 7A). In contrast, 6/12 peptide-vaccinated Rag^-/-^ mice remained tumor free (Fig 7A). Surprisingly, however, only 2/12 Rag^-/-^ mice rejected EG7 lymphomas after rIL-7 therapy (Fig 7A). Importantly, this was not due to impaired DC expansion in rIL-7-treated Rag^-/-^ mice (Fig 7C). Hence, the protective effect of peptide vaccination was blunted by rIL-7.

Correlating with their comparably low frequency (Fig 4B) and KLRG-1^hi^IFN-γ^lo^ phenotype (Fig 4E and 4H), SIINFEKL-induced T_M_ cells rejected EG7 tumors only in 2/12 Rag^-/-^IL-7R^-/-^ mice (Fig 7B). After rIL-7 therapy only 1/12 Rag^-/-^IL-7R^-/-^ mice remained tumor free (Fig 7B). In summary, the protective effect of peptide vaccination relied on host IL-7R signaling and was blunted by rIL-7 therapy.

## Discussion

The major goal of our study was to clarify whether and how host IL-7R signaling contributes to rIL-7-driven anti-tumor CD8^+^ T cell responses. For this purpose, SIINFEKL-specific CD8^+^ OT-I cells were transferred into Rag^-/-^ and Rag^-/-^IL-7R^-/-^ mice, which were then treated with rIL-7. As shown in Fig 1A, OT-I-reconstituted Rag^-/-^ mice receiving IL-7 therapy rejected SIINFEKL-expressing EG7 lymphoma cells. This was not the case for Rag^-/-^IL-7R^-/-^ mice demonstrating that the success of rIL-7 therapy is dependent on host IL-7R expression in our experimental system. However, CD8^+^ T cell expansion and differentiation were largely independent of host IL-7R signaling. Irrespective of host IL-7R expression nearly identical numbers of OT-I cells were recovered from spleens after rIL-7 treatment. Similarly, the differentiation of CD8^+^ T_M_ with a CD127^hi^Bcl-2^hi^CD62L^hi^KLRG-1^lo^IFN-γ^hi^ phenotype was induced in rIL-7-treated Rag^-/-^ and Rag^-/-^IL-7R^-/-^ mice. Hence, our data show that rIL-7-induced CD8^+^ T cell expansion and subsequent T_M_ differentiation are not affected by host IL-7R expression and related differences in IL-7 availability (Fig 1D). In agreement with previous studies [21], the beneficial effects of rIL-7 on CD8^+^ T cell function, differentiation and survival appear to result mainly from IL-7R signaling in CD8^+^ T cells. Nevertheless, it is important to stress that effective CD8^+^ T cell expansion and differentiation do not necessarily correlate with tumor rejection. This conclusion is supported by the fact that rIL-7-induced OT-I expansion and subsequent T_M_ differentiation occurred efficiently in Rag^-/-^IL-7R^-/-^ mice while tumor rejection failed. This finding emphasizes the importance of IL-7-responsive host cells for rIL-7-assisted ATT in our model system.

Dendritic cells promote CD8^+^ T cell responses under lymphopenic conditions [40], cross-present tumor-derived antigens [41] and expand in response to rIL-7 treatment [24], which promotes T-DC interactions [42]. Additionally, rIL-7 stimulates myelopoiesis and the subsequent accumulation of CD11b^+^ cells [25], which can cross-present tumor antigens and promote CD8^+^ T cell-mediated tumor rejection [43]. After rIL-7 treatment, DCs and granulocytes accumulated only in IL-7R-competent mice. However, this accumulation was not required for expansion and functional maturation of OT-I cells but correlated positively with tumor rejection. This suggests that rIL-7-expanded DCs and granulocytes support CD8^+^ T cell responses in the late effector phase rather than in the early phase after adoptive transfer.

IL-7R signaling in combination with TCR stimulation boosts CD8^+^ T cell responses in multiple experimental systems. TCR signal strength and the timing of IL-7R signaling appear to be important to achieve optimal IL-7 effects [44]. To generate maximum CD8^+^ T cell responses, we reconstituted Rag^-/-^ and Rag^-/-^IL-7R^-/-^ mice with OT-I cells and vaccinated them with SIINFEKL. Unlike after rIL-7 treatment alone, host IL-7R signaling was crucial for maximum OT-I expansion in response to vaccination and rIL-7. Additionally, OT-I cells up-regulated CD127 and down-modulated PD-1 in a host-IL-7R-dependent fashion. This was not the case for all other markers tested. The host-dependent modulation of CD8^+^ T cell differentiation was confirmed in BM chimeras. Surprisingly, we identified IL-7R^+^ radio-resistant host cells as major regulators of CD8^+^ T cell expansion and differentiation. For maximum levels of CD127 expression and restriction of KLRG-1, CD132, Bcl-2, CD8, and PD-1, IL-7R expression by radio-resistant host cells was sufficient. CD127 expression is modulated by multiple intracellular signaling events in T cells. Whether non-BM-derived host cells affect these or other signaling pathways remains open.

Besides OT-I expansion and differentiation, IL-7R^+^ non-BM-derived cells also controlled granulocyte and DC expansion. Even if donor BM was devoid of the IL-7R, its expression by radio-resistant cells supported the expansion of CD11b^+^ cells and DCs. However, IL-7R expression by BM-derived cells was largely sufficient to rescue the expansion of CD11b^+^ cells and CD8^-^ DCs. Surprisingly, this was not the case for CD8^+^ DCs. Their accumulation depended more on IL-7R expression by non-BM- than by BM-derived cells. Together, we demonstrate that IL-7R signaling in BM- and non-BM-derived cells contributes to rIL-7-driven DC expansion. This might help to reconcile apparently conflicting results regarding the relative importance of cell autonomous IL-7R signaling for DC generation obtained in different experimental systems [24,45].

Fibroblastic reticular cells (FRCs) and lymphoid endothelial cells (LECs) are major sources of IL-7 in secondary lymphoid organs [46]. Furthermore, other cells of non-BM origin such as intestinal epithelial cells (IECs) [47], keratinocytes [48], hepatocytes [49] and fibroblasts [50] were shown to produce IL-7 *in vivo*. The maintenance of CD8^+^ T cell homeostasis requires IL-7R signaling in CD8^+^ T cells which is triggered by IL-7 from non-BM derived cells [20]. However, only little is known about the consequences of IL-7R signaling in non-BM-derived cells and subsequent immune modulation [51]. As we have shown in S2 Fig, the application of rIL-7 leads to the systemic down-modulation of *il-7* gene activity supporting previous reports demonstrating that *il-7* gene activity is regulated in an IL-7-mediated negative feedback loop [4,12]. Given that IL-7R signaling modulates gene expression profiles in multiple non-BM-derived cell types [4,12,26], long-term rIL-7 therapy would not only affect immune cell but also non-immune cell homeostasis. For instance, prolonged IL-7 overabundance promotes IEC expansion, survival and subsequent alterations in intestinal physiology [12]. Since many cell types of non-BM-derived origin can express the IL-7R [31], the local down regulation of endogenous IL-7 production and alterations in tissue homeostasis might be as yet underestimated side effects of rIL-7 therapy. Whether i) non-BM-derived cells located in the tumor stroma and/or other tissues are the main targets of rIL-7, and ii) whether rIL-7 signaling in such cells promotes or suppresses rIL-7-assisted ATT remains to be shown.

IL-7R^+^ host cells appear to promote antigen-dependent CD8^+^ T cell function also in an IL-7-independent fashion. For example, successful peptide vaccination and subsequent tumor rejection, in the absence of rIL-7 treatment, strictly required host IL-7R expression (Fig 7A and 7B). Importantly, however, peptide vaccination blocked the therapeutic effect of rIL-7 (Figs 1A and 7A) in Rag^-/-^ mice although IL-7R-dependent DC expansion was normal (Fig 7C). It has been reported that TCR signaling can interfere with the beneficial effects of IL-7 on T cells [52]. Hence, impaired tumor rejection in rIL-7-treated, peptide vaccinated Rag^-/-^ mice might have been a result of altered T cell rather than host cell function.

In summary, our data provide evidence for the complex interplay between IL-7R^+^ host and CD8^+^ T cells in the course of anti-tumor CD8^+^ T cell responses. While productive host-CD8^+^ T cell interactions can be promoted by rIL-7 therapy, the inappropriate combination with other immune stimuli can cause adverse effects.

## Supporting Information

S1 Fig Lymphopenia-induced T cell proliferation (LIP) is independent of host IL-7R signaling. (A, B) Rag^-/-^, Rag^-/-^IL-7R^-/-^ and WT mice were reconstituted with 1 x 10^6^ CD8^+^CD90.1^+^ OT-I T cells. Spleens were analyzed by flow cytometry 21–27 days later. Shown are (A) relative fluorescence intensities for CD44 on CD8^+^CD90.1^+^ OT-I T cells. (B) Cell numbers were normalized to the mean values determined in treated Rag^-/-^ mice and are shown as relative (rel.) values. Data (±SEM) from 3 independent experiments with a total of 9–19 mice/group are shown.
(EPS)

S2 Fig IL-7 administration decreases host *il-7* expression. (A, B) IL- representative mouse before (0h) and 24 hours (24h) after IL-7 treatment are shown. (B) BLI levels measured at 24h were calculated in relation to those determined before treatment and relative BLI (rel. BLI) values were calculated. Shown are pooled data (± SEM) of 2 experiments with a total of 6 mice. Statistical analysis was performed using Wilcoxon matched-pairs signed rank test.
(EPS)
